# Embryonic type Na^+^ channel β-subunit, *SCN3B* masks the disease phenotype of Brugada syndrome

**DOI:** 10.1038/srep34198

**Published:** 2016-09-28

**Authors:** Shinichiro Okata, Shinsuke Yuasa, Tomoyuki Suzuki, Shogo Ito, Naomasa Makita, Tetsu Yoshida, Min Li, Junko Kurokawa, Tomohisa Seki, Toru Egashira, Yoshiyasu Aizawa, Masaki Kodaira, Chikaaki Motoda, Gakuto Yozu, Masaya Shimojima, Nozomi Hayashiji, Hisayuki Hashimoto, Yusuke Kuroda, Atsushi Tanaka, Mitsushige Murata, Takeshi Aiba, Wataru Shimizu, Minoru Horie, Kaichiro Kamiya, Tetsushi Furukawa, Keiichi Fukuda

**Affiliations:** 1 Department of Cardiology, Keio University School of Medicine, Japan; 2 Department of Bio-informational Pharmacology, Medical Research Institute, Tokyo Medical and Dental University, Japan; 3 Department of Cardiovascular Research, Research Institute of Environmental Medicine, Nagoya University, Japan; 4 Department of Molecular Physiology, Nagasaki University Graduate School of Biomedical Sciences, Nagasaki, Japan; 5 Division of Gene Therapy, Research Center of Genome Medicine, Saitama Medical University, Japan; 6 Department of Laboratory Medicine, Keio University School of Medicine, Tokyo, Japan; 7 Division of Arrhythmia and Electrophysiology, Department of Cardiovascular Medicine, National Cerebral and Cardiovascular Center, Osaka, Japan; 8 Department of Cardiovascular Medicine, Nippon Medical School, Tokyo, Japan; 9 Department of Cardiovascular Medicine, Shiga University of Medical Science, Shiga, Japan

## Abstract

*SCN5A* is abundant in heart and has a major role in *I*_*Na*_. Loss-of-function mutation in *SCN5A* results in Brugada syndrome (BrS), which causes sudden death in adults. It remains unclear why disease phenotype does not manifest in the young even though mutated SCN5A is expressed in the young. The aim of the present study is to elucidate the timing of the disease manifestation in BrS. A gain-of-function mutation in *SCN5A* also results in Long QT syndrome type 3 (LQTS3), leading to sudden death in the young. Induced pluripotent stem cells (iPSCs) were generated from a patient with a mixed phenotype of LQTS3 and BrS with the E1784K *SCN5A* mutation. Here we show that electrophysiological analysis revealed that LQTS3/BrS iPSC-derived cardiomyocytes recapitulate the phenotype of LQTS3 but not BrS. Each β-subunit of the sodium channel is differentially expressed in embryonic and adult hearts. *SCN3B* is highly expressed in embryonic hearts and iPSC-derived cardiomyocytes. A heterologous expression system revealed that *I*_*Na*_ of mutated SCN5A is decreased and *SCN3B* augmented *I*_*Na*_ of mutated SCN5A. Knockdown of *SCN3B* in LQTS3/BrS iPSC-derived cardiomyocytes successfully unmasked the phenotype of BrS. Isogenic control of LQTS3/BrS (corrected-LQTS3/BrS) iPSC-derived cardiomyocytes gained the normal electrophysiological properties.

Na^+^ channels initiate excitation in cardiomyocytes by opening to produce a large inward depolarizing ionic current (*I*_Na_). Once activated, the channels inactivate rapidly, extinguishing *I*_Na_ within 10 ms. Mutations in *SCN5A*, which encodes the α-subunit of cardiac Na^+^ channels, cause various types of hereditary arrhythmia syndromes, including Long QT syndrome type 3 (LQTS3) and Brugada syndrome (BrS) among others^1^,^2^,^3^. BrS is associated with a high risk of sudden cardiac death in adults, and less frequently in infants and children^4^. *In vitro* functional characterization of the *SCN5A* mutants of BrS demonstrated the loss-of-function phenotypes (hyperpolarizing shift of inactivation, a depolarizing shift of activation, and a reduction in peak sodium current) expected to cause BrS. It remains unclear why loss-of-function mutations in *SCN5A* primarily affect adults and whether adolescent disease manifestation would be regulated by cell autonomous manner.

Recent studies report that, following the generation of human induced pluripotent stem cells (iPSCs) from patients with hereditary diseases, the differentiation of these iPSCs into various types of cells, including cardiomyocytes, can reproduce the disease phenotype^5^,^6^,^7^,^8^,^9^,^10^,^11^. These disease models based on iPSC-derived cells could provide a tool with which to examine disease pathogenesis and to search for treatments^12^,^13^. However, the function and gene expression profile of iPSC-derived cells is similar to that of embryonic rather than adult cells; this is also true of iPSC-derived cardiomyocytes, which exhibit immature electrophysiological and contractile functions as well as immature gene expression profiles for ion channels and contractile proteins^14^,^15^. The phenotype of many hereditary diseases develops later in life. In terms of disease modeling using iPSCs, it is not known whether patient-specific iPSC-derived cells will recapitulate the phenotype of these late-onset arrhythmic diseases. If not, which factor/s are responsible for the failure of late-onset disease manifestation: extrinsic environmental factors and the resultant epigenetic changes, intrinsic cell factors, or others? From this viewpoint, it is of interest to examine iPSC-derived cardiomyocytes from patients with late-onset arrhythmic diseases.

To model BrS using iPSCs, we generated iPSCs from a patient with mixed phenotype of LQTS3 and BrS. Mutations in *SCN5A* leading to LQTS3 produce gain-of-function defects by disrupting Na^+^ channel inactivation, thereby causing a small persistent *I*_Na_ during the action potential plateau^16^,^17^. Clinically, LQTS is associated with sudden death in the young, particularly in the case of LQTS3, which sometimes affects infants and children^18^,^19^. Interestingly, there are single mutations at *SCN5A* that induce a mixed phenotype of LQTS3 and BrS^20^,^21^,^22^. *In vitro* functional characterization of these *SCN5A* mutants demonstrated that they exhibited loss-of-function phenotypes expected to cause BrS concurrently with a gain-of-function phenotype, which accounts for LQTS3. It is not known why loss-of-function mutations primarily affect adults. From this viewpoint, it is of interest to examine whether iPSC-derived cardiomyocytes from patients with mixed phenotypes of LQTS3 and BrS (LQTS3/BrS iPSC-derived cardiomyocytes) simulate the phenotype of LQTS3 frequently associated with young age in addition to that of BrS, which is frequently associated with adulthood, similar to that seen in patients. To this end, the aims of the present study were to generate iPSCs from a patient with mixed phenotypes of LQTS3 and BrS, to examine which phenotype/s were manifested in patient-specific iPSC-derived cardiomyocytes, and to elucidate the mechanisms of temporal phenotype manifestation in BrS. Our data show that LQTS3/BrS iPSC-derived cardiomyocytes exhibit phenotypes similar to LQTS3, but not BrS. The temporal expression pattern of the fetal Na^+^ channel β-subunit *SCN3B* affects the manifestation of BrS phenotypes in LQTS3/BrS iPSC-derived cardiomyocytes.

## Results

### Generation of LQTS3/BrS iPSCs and LQTS3/BrS iPSC-derived cardiomyocytes

**Figure 1:**
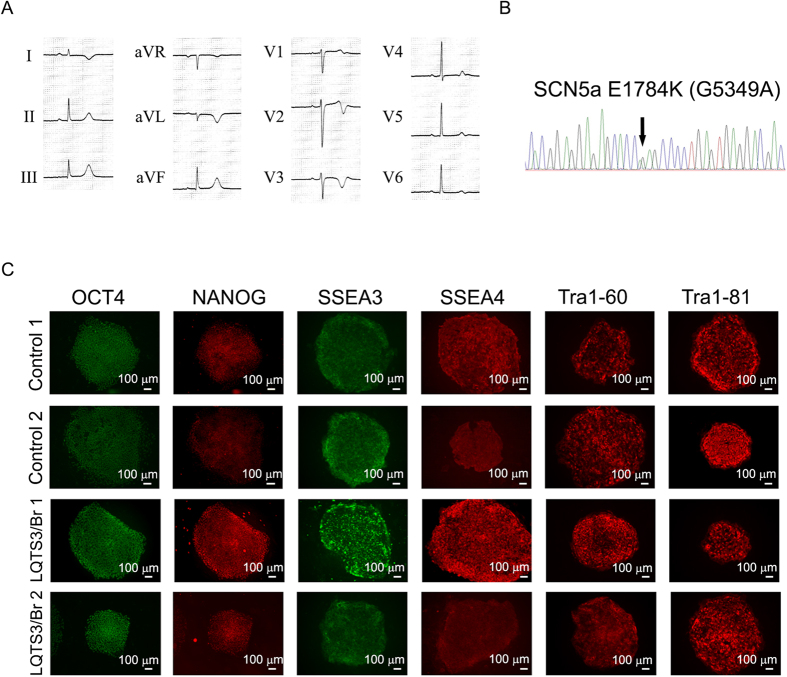
Generation of iPSCs from two healthy volunteers and a patient with LQTS3 and BrS. (**A**) Electrocardiogram from the patient during sinus rhythm. QTc 520 ms. (**B**) Sequence analysis of genomic *SCN5A* in the patient. (**C**) Immunofluorescence staining for stem cell markers (OCT4, NANOG, SSEA3, SSEA4, Tra1-60 and Tra1-81) in two control and two LQTS3/BrS iPSC colonies.

A 20-year-old man was admitted with sudden cardiac arrest that occurred while he was driving a car. The patient subsequently underwent successful resuscitation using an automated external defibrillator, the data from which showed ventricular fibrillation. The surface electrocardiogram showed a significantly prolonged QT interval and QT interval corrected for heart rate (i.e. QTc; Fig. 1A). The patient had no family history of previous syncope episodes or significant QT interval abnormalities. The pilsicainide administration test induced coved-type ST elevation in the V1 and V2 precordical leads. Because the clinical findings on syncope, electrocardiogram morphology, and drug testing suggested a mixed phenotype of LQTS3 and BrS, we genotyped the patient and identified the *SCN5A* E1784K (G5349A) mutation (Fig. 1B), which was shown previously to be associated with the mixed phenotype of LQTS3 and BrS.

To examine which phenotypes can be recapitulated in patient-specific iPSC-derived cardiomyocytes and elucidate the mechanisms of temporal phenotype manifestation in LQTS3 and BrS, we generated iPSCs from this patient with a mixed LQTS3/BrS phenotype. To generate iPSCs, we used dermal fibroblasts from the patient and two volunteers, and reprogrammed these cells using retrovirus-mediated gene transfer of *SOX2*, *OCT4* (also known as *POU5F1*), *KLF4*, and *MYC*^23^. Several clones were generated, expanded and stored. All iPSC lines showed typical iPSC morphology and expressed human pluripotency markers (Fig. 1C). To examine pluripotency, LQTS3/BrS iPSCs were injected into NOD-SCID mice. The teratomas derived from the injected LQTS3/BrS iPSCs contained the cell derivatives of all three germ layers, such as cartilage, intestine, and neural tissue (Supplementary Figure 1A). An embryoid body (EB) culture system was used to differentiate the iPSCs into cardiomyocytes^24^,^25^,^26^,^27^. Immunofluorescence staining for dissociated cardiomyocytes showed clear immunopositivity for cardiac-specific gene products in control and LQTS3/BrS iPSC-derived cardiomyocytes (Supplementary Figure 1B).

### LQTS3/BrS iPSC-derived cardiomyocytes recapitulate the phenotype of LQTS

**Figure 2:**
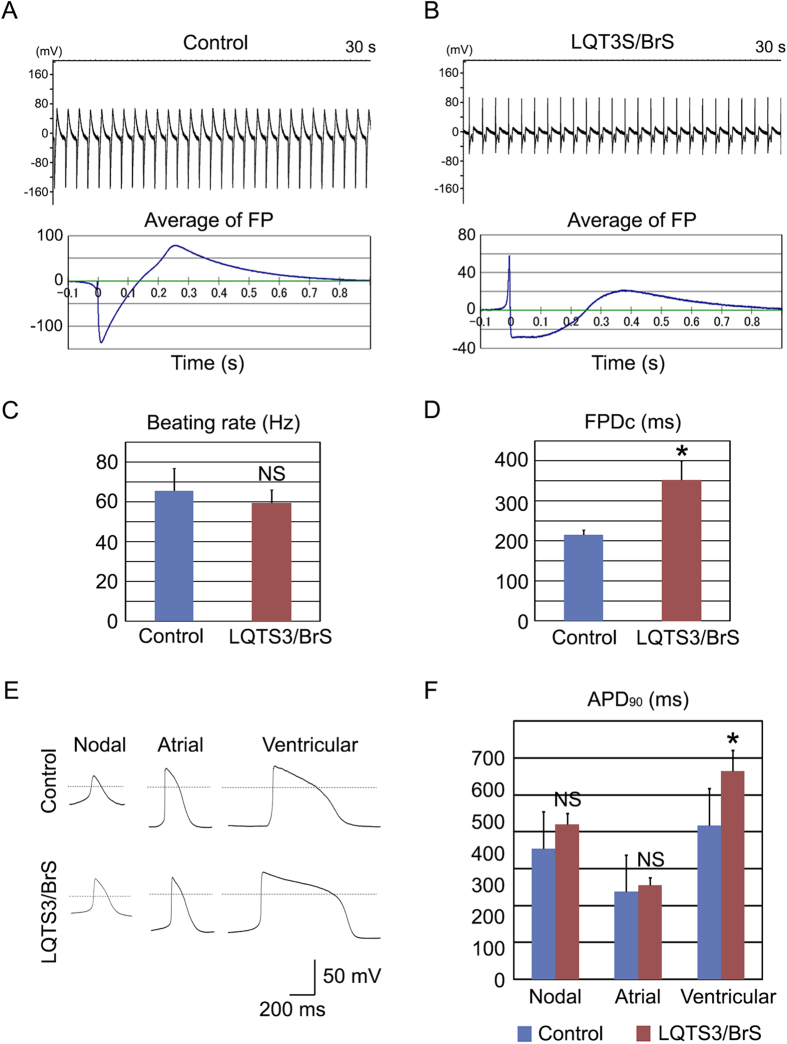
Electrophysiological features of LQTS3/BrS iPSC-derived cardiomyocytes. (**A**,**B**) Representative MEA recordings from control and LQTS3/BrS iPSC-derived beating EBs. FP, field potential. (**C**) Rate of beating of control and LQTS3/BrS iPSC-derived EBs. (**D**) Average corrected field potential duration (cFPD) recorded with the MEA in control (n = 6) and LQTS3/BrS iPSC-derived (n = 7) beating EBs. (**E**) Representative action potentials of control and LQTS3/BrS iPSC-derived cardiomyocytes showing nodal-, atrial- and ventricular-type morphology, recorded in spontaneous firing cardiomyocytes. The dashed line indicate 0 mV. (**F**) Statistical parameters of action potential duration at 90% repolarization (APD_90_) obtained from control and LQTS3/BrS iPSC-derived cardiomyocytes exhibiting nodal- (n = 6 and 19, respectively), atrial- (n = 6 and 4, respectively) and ventricular-type (n = 10 and 7, respectively) morphology. Where appropriate, data are given as the mean ± SEM. *P < 0.05 compared with control.

To investigate the electrophysiological properties, a multielectrode array (MEA) system was used that enabled measurement of the surface electrogenic activities of cardiomyocytes and could be adapted to automatic high-throughput systems^28^. The MEA analyses revealed that control and LQTS3/BrS iPSC-derived EBs showed similar rhythmic electrical activity and rate of spontaneous beating (Fig. 2A–C). The field potential duration (FPD) in MEA analysis is analogous to the QT interval in a surface electrocardiogram^28^. The cFPD (normalized for beating frequency) of LQTS3/BrS iPSC-derived EBs was significantly longer than that of controls (Fig. 2D), suggesting that iPSC-derived cardiomyocytes from LQTS3/BrS iPSC recapitulated the phenotype of LQTS. To confirm that the FPD reflects action potential duration (APD), we measured the action potentials of control and LQTS3/BrS iPSC-derived cardiomyocytes directly using the patch-clamp technique. Day 30 beating EBs were selected manually and were dispersed enzymatically into single cells. The dispersed single cardiomyocytes were cultured for 3 days before analysis. Rhythmic beating was detected in both control and LQTS3/BrS iPSC-derived cardiomyocytes. The action potentials recorded resembled those of the sinus node (i.e. relatively shallow resting membrane potential, slow diastolic depolarization, and relatively long APD), the atria (i.e. relatively deep resting membrane potential, sharp systolic depolarization, and relatively short APD), and the ventricle (i.e. relatively deep resting membrane potential, sharp systolic depolarization, and relatively long APD). There were no significant differences in the APD of nodal or atrial types of cardiomyocytes between the control and LQTS3/BrS iPSC-derived groups (Fig. 2E,F; Supplementary Table). However the action potential of ventricular type cardiomyocytes exhibited a significantly greater APD_90_ for LQTS3/BrS compared with control iPSC-derived cardiomyocytes. These data suggest that LQTS3/BrS iPSC-derived cardiomyocytes recapitulate the LQTS phenotype.

### LQTS3/BrS iPSC-derived cardiomyocytes recapitulate the phenotype of LQTS3, but not BrS

**Figure 3:**
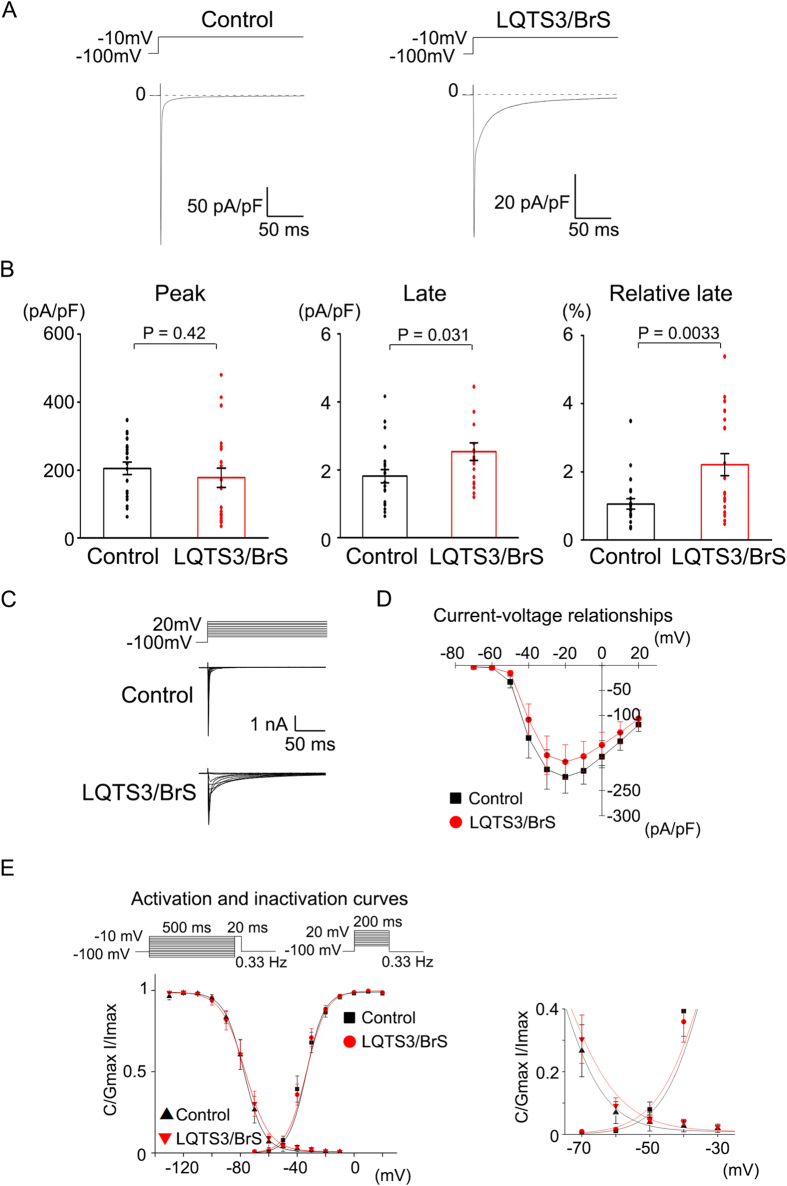
Electrophysiological features of LQTS3/BrS iPSC-derived cardiomyocytes, as determined by patch-clamp analyses. (**A**) Representative current traces of baseline in control and LQTS3/BrS iPSC-derived cardiomyocytes. (**B**) The average and data plots of peak current, late current, and relative late current in control (n = 22) and LQTS3/BrS iPSC-derived cardiomyocytes (n = 21). (**C**) Representative current traces of baseline in control and LQTS3/BrS iPSC-derived cardiomyocytes. The pulse protocol is shown in the upper panel. (**D**) Average current–voltage relationship for peak current in control (n = 9) and LQTS3/BrS iPSC-derived cardiomyocytes (n = 10). (**E**) Average voltage-dependent activation and inactivation curves in control (n = 9, 4 respectively) and LQTS3/BrS iPSC-derived cardiomyocytes (n = 10, 7 respectively). Where appropriate, data are given as the mean ± SEM.

Although it has been reported previously that the *SCN5A* E1784K mutation exhibits the mixed phenotype of LQTS3 and BrS in a heterologous overexpression system (i.e. a human mutant gene in human non-cardiomyocytes or a human mutant gene in non-human cardiomyocytes)^22^, it was not clear whether we would be able to recapitulate the phenotype of BrS in LQTS3/BrS iPSC-derived cardiomyocytes with the *SCN5A* E1784K mutation. We examined the sodium current in human cardiomyocytes derived from control iPSCs and iPSCs harboring the *SCN5A* E1784K mutation by patch-clamp analysis (Fig. 3A). Although there was no significant difference in peak sodium current between the control and LQTS3/BrS iPSC-derived cardiomyocytes, the late sodium current and relative late sodium current were significantly increased in LQTS3/BrS iPSC-derived cardiomyocytes (Fig. 3B). These data indicate that LQTS3/BrS iPSC-derived cardiomyocytes recapitulate the phenotype of LQTS3 in terms of late sodium current augmentation. However, the peak I-V relationship curve did not differ significantly between the control and LQTS3/BrS iPSC-derived cardiomyocytes (Fig. 3C,D). Nor were there any significant differences in the voltage-dependent activation and inactivation curves between the two groups (Fig. 3E). Both the negative shift of steady-state inactivation and the positive shift of activation reduced channel availability and conductance, which were predicted to decrease Na current in BrS. But in the present study the LQTS3/BrS iPSC-derived cardiomyocytes did not exhibit any significant differences in steady state inactivation. These data indicate that LQTS3/BrS iPSC-derived cardiomyocytes recapitulate the LQTS3 phenotype, but not the BrS phenotype.

### Effects of *SCN5A* plus β-subunits on sodium currents in tsA-201 cells

**Figure 4:**
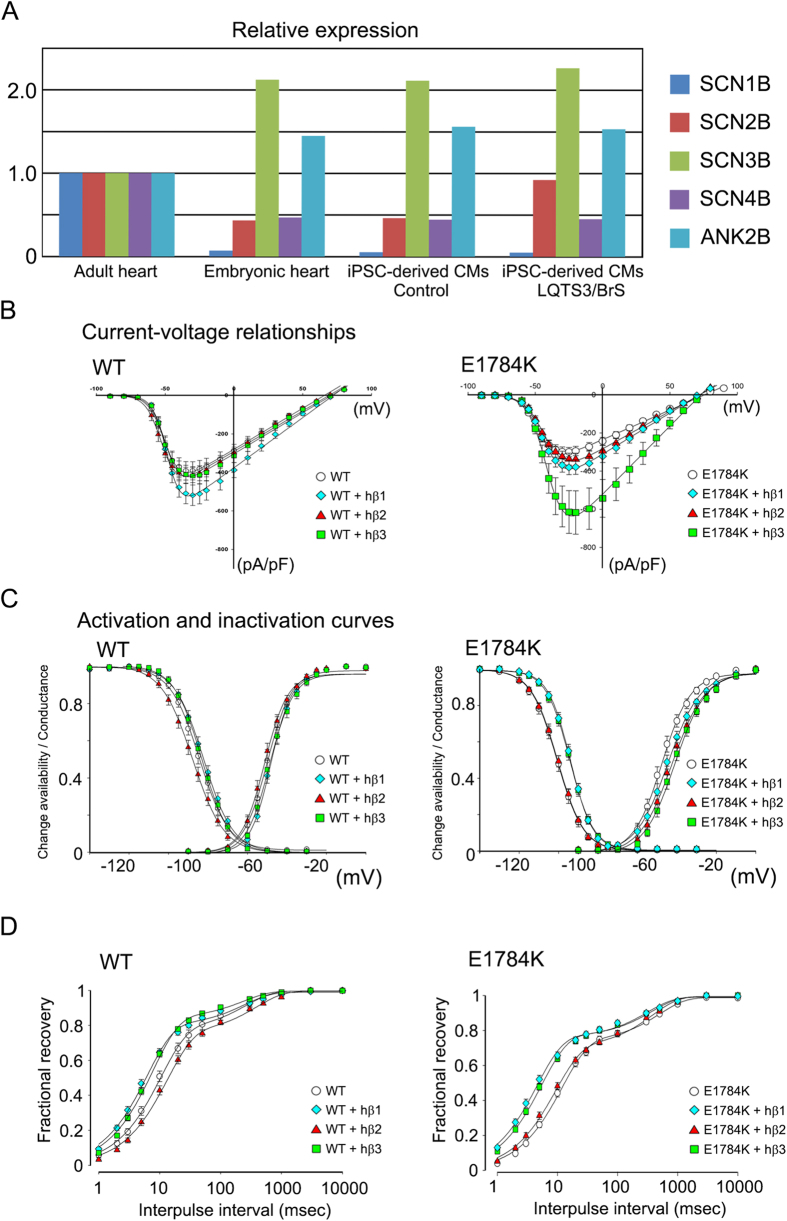
The subunit compound dependent distinct Na^+^ current of WT and E1784 SCN5A in tsA-201 cells. (**A**) Quantitative RT-PCR analyses for *SCN1B*, *SCN2B*, *SCN3B*, *SCN4B* and *ANK2B* in adult human heart (mixed sample from three men aged 30–39 years), embryonic heart (mixed human sample from 34 male and female embryos at 12–31 weeks gestation), control-iPSC-derived-cardiomyocytes and LQTS3/BrS-iPSC-derived cardiomyocytes. (**B**) Current–voltage relationships for peak current during the test depolarization pulse in tsA-201 cells with either WT *SCN5A* or the *SCN5A* E1784K mutation alone (n = 15 and 11, respectively) or with the *SCN1B* (n = 17 for both), *SCN2B* (n = 20 and 17, respectively), or *SCN3B* (n = 17 for both) genes. (**C**) Voltage dependency of activation and inactivation curves in tsA-201 cells with either WT *SCN5A* or the *SCN5A* E1784K mutation alone (n = 11 and 15, respectively) or with the *SCN1B* (n = 17 for both), *SCN2B* (n = 18 and 17, respectively) or *SCN3B* (n = 17 for both) genes. (**D**) Fractional recovery from inactivation of tsA-201 cells with either WT *SCN5A* or the *SCN5A* E1784K mutation alone (n = 15 and 11, respectively) or with the *SCN1B* (n = 21 and 19, respectively), *SCN2B* (n = 19 and 17, respectively) or *SCN3B* (n = 19 and 17, respectively) genes. Where appropriate, data are given as the mean ± SEM.

Next we compared the expression profiles of the β-subunits of sodium channels among adult hearts, embryonic hearts, and iPSC-derived cardiomyocytes in human (Fig. 4A). Interestingly, the gene expression profiles of iPSC-derived cardiomyocytes were quite comparable to those of embryonic hearts. High *SCN3B* expression was detected in embryonic compared with adult hearts. To examine the role of β-subunits in sodium current, we used a heterologous gene expression system in tsA-201 cells. Electrophysiological characterization of tsA-201 cells, expressing *SCN1B*, *SCN2B*, or *SCN3B*, with exogenous wild type (WT) or mutated *SCN5A* (E1784K) genes revealed that cells with either the exogenous WT or E1784K had typical *I*_Na_ currents. Furthermore, in WT cells, the I-V relationship was affected by the type of β-subunit expressed; specifically, the introduction of *SCN1B* increased the density of *I*_Na_, whereas that of *SCN2B* and *SCN3B* did not (Fig. 4B). In contrast, in E1784K cells, the density of *I*_Na_ was increased following the introduction of *SCN3B*, but not *SCN1B* and *SCN2B* (Fig. 4B). Previous studies have reported that a loss-of-function mutation in *SCN5A* results in BrS^29^,^30^. In the present study, *SCN3B* increased *I*_Na_ current density in E1784K cells, indicating that high *SCN3B* expression in the embryonic heart and iPSC-derived cardiomyocytes may explain why the BrS phenotype is masked in young patients. Voltage-dependent activation and inactivation curves did not differ significantly in WT cells with either *SCN1B*, *SCN2B*, or *SCN3B* (Fig. 4C). In contrast, a positive shift in the inactivation, but not activation, curve was seen in E1784K cells with *SCN1B* or *SCN3B* (Fig. 4C). In addition, the fraction of channels entering the slow inactivated state and the rate of development of slow inactivation (fractional recovery) differed in WT cells with either *SCN1B* or *SCN3B*, but not *SCN2B* (Fig. 4D). Fractional recovery also differed in E1784K cells with either *SCN1B* or *SCN3B*, but not *SCN2B* (Fig. 4D). These data suggest that the expression of β-subunits would affect the characteristics of *I*_Na_.

### Effects of SCN3B knockdown on the BrS phenotype in LQTS3/BrS iPSC-derived cardiomyocytes

**Figure 5:**
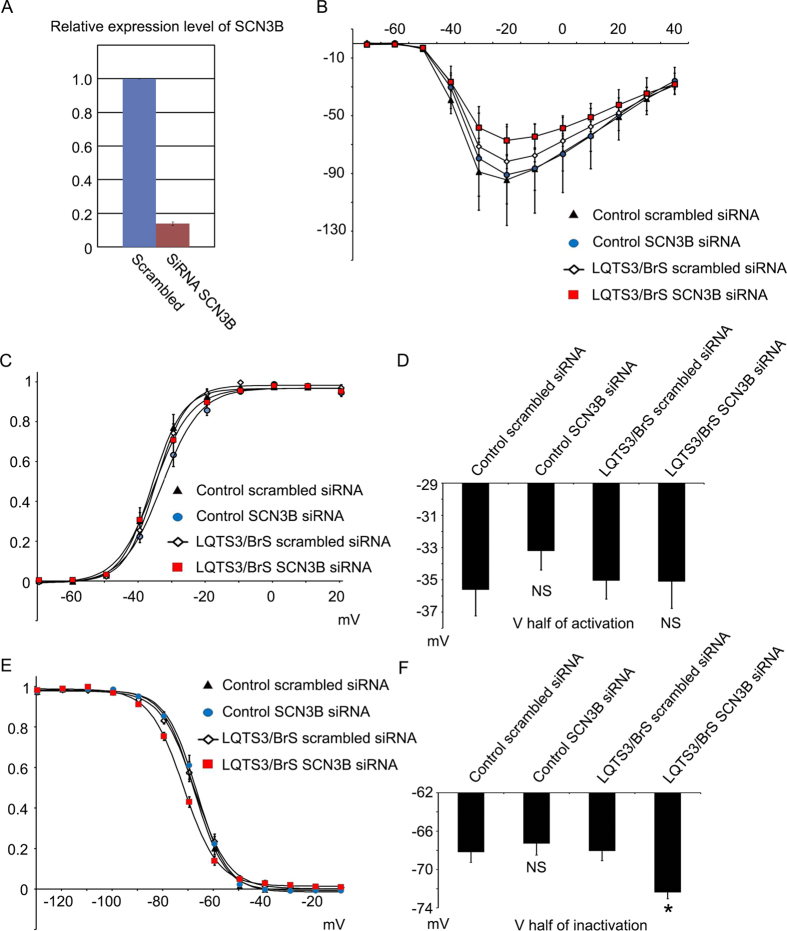
Electrophysiological features of LQTS3/BrS iPSC-derived cardiomyocytes following *SCN3B* knockdown, as determined by patch-clamp analyses. (**A**) Quantitative RT-PCR analyses of *SCN3B* in iPSC-derived cardiomyocytes transfected with either scrambled siRNA (n = 3) or siRNA for *SCN3B* (n = 3). (**B**) Average current–voltage relationship for peak current in control and LQTS3/BrS iPSC-derived cardiomyocytes transfected with scrambled siRNA or siRNA for *SCN3B* (n = 10 for all groups). (**C**,**E**) Average voltage dependency of activation (**C**) and inactivation (**E**) in control and LQTS3/BrS iPSC-derived cardiomyocytes transfected with scrambled siRNA or siRNA for *SCN3B*. (**D**,**F**) V_½_ of activation (**D**) and inactivation (**F**) in control and LQTS3/BrS iPSC-derived cardiomyocytes transfected with scrambled siRNA (n = 9 and 13, respectively) or siRNA for *SCN3B* (n = 10 and 11, respectively). Where appropriate, data are given as the mean ± SEM. *P < 0.05 compared with control.

*SCN3B* is highly expressed in embryonic hearts and iPSC-derived cardiomyocytes, and augments *I*_Na_ in tsA-201 cells expressing mutated *SCN5A*. These observations suggest that *SCN3B* may be involved in the manifestation of disease phenotype in BrS. To elucidate whether high expression of *SCN3B* masked the phenotype of BrS, *SCN3B* was knocked down by siRNA in control and LQTS3/BrS iPSC-derived cardiomyocytes (Fig. 5A), followed by electrophysiological characterization. The knockdown of *SCN3B* in control had no significant effect on *I*_Na_ density in the I-V curves. But the knockdown of *SCN3B* in LQTS3/BrS showed decreased *I*_Na_ density in the I-V curves (Fig. 5B). Furthermore, the knockdown of *SCN3B* in control and LQTS3/BrS had no effect on the voltage dependence of steady state activation (Fig. 5C,D). However, a negative shift in the voltage dependence of steady state inactivation was seen following *SCN3B* knockdown in LQTS3/BrS (Fig. 5E,F). These data indicate that the decrease of *SCN3B* successfully unmasks the phenotype of BrS in LQTS3/BrS iPSC-derived cardiomyocytes

### Corrected-LQTS3/BrS iPSC-derived cardiomyocytes diminish the phenotype of LQTS3

**Figure 6:**
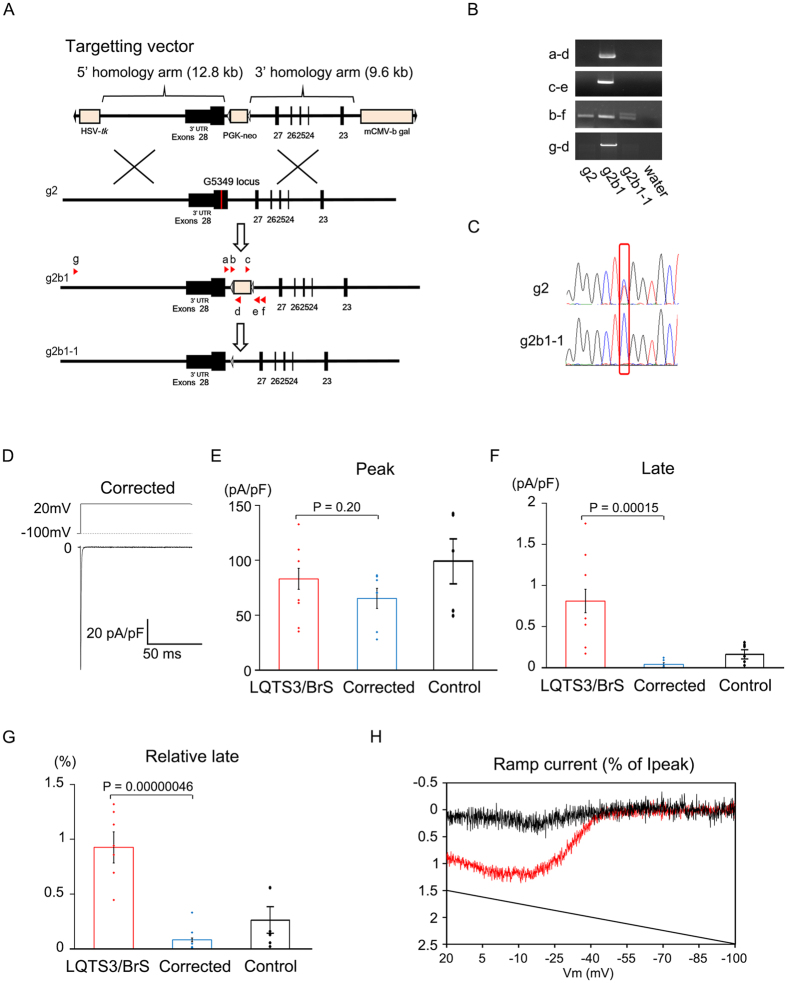
Electrophysiological features of corrected-LQTS3/BrS iPSC-derived cardiomyocytes, as determined by patch-clamp analyses. (**A**) Schematic illustrations of the *SCN5A* gene correction implemented in the LQTS3/BrS iPSCs using HDAdVs. HDAdV, helper-dependent adenoviral vector; HSV-*tk*, herpes simplex virus thymidine kinase gene cassette; PGK-*Neo*, neomycin-resistance gene cassette; white triangles, loxP sites; mCMV-β*-gal*,β galactosidase gene cassette; red line, exon 28 of *SCN5A* containing the E1784K mutation. (**B**) PCR analysis confirmed that recombination occurred at the *SCN5A* locus. Products of 311 bp, 314 bp, 277 bp or 375 bp (the footprint of the targeting vector included) and 13156 bp were obtained using primers a-d, c-e, b-f and g-d (red arrowheads in A), respectively. (**C**) Sequence analysis of the *SCN5A* genes in each iPSC line. (**D**) Representative current trace of baseline in corrected-LQTS3/BrS iPSC-derived cardiomyocytes. (**E**–**G**). The average and data plots of peak current, late current, and relative late current in LQTS3/BrS (n = 13), corrected-LQTS3/BrS iPSC-derived cardiomyocytes (n = 7) and control-iPSC-derived cardiomyocytes (n = 6). (**H**) Averaged normalized LQTS3/BrS (n = 13) and corrected-LQTS3/BrS (n = 7) window currents obtained with a 100 ms repolarizing negative voltage ramp from +20 and −100 mV (1.2 mV/ms), normalized to the peak current recorded in the same cell.

To address whether the *SCN5A* E1784K mutation is necessary and/or sufficient for observed electrophysiological characteristics, we generated isogenic control of LQTS3/BrS (corrected-LQTS3/BrS)-iPSCs using helper-dependent adenoviral vector (HDAdV). A wild-type *SCN5A ge*ne in a BAC clone, with a *Neo* cassette introduced in the intron, was inserted into an HDAdV (Fig. 6A). The genome sequence of LQTS3/BrS-iPSCs (g2) was replaced with the wild-type SCN5A gene through homologous recombination (g2b1), followed by the removal of the *Neo* cassette by Cre recombinase (g2b1-1) (Fig. 6A). We confirmed successful gene editing in corrected-LQTS3/BrS iPSCs (Fig. 6B,C, Supplementary Figures 2A and 4B). The APs of corrected-LQTS3/BrS iPSC-derived cardiomyocytes resembled those of the sinus node, the atria, and the ventricle (Supplementary Figure 2C). There were no significant differences in the APD of nodal or atrial types of cardiomyocytes between the LQTS3/BrS and corrected-LQTS3/BrS (Supplementary Figure 2D, Supplementary Table). However the action potential of ventricular type cardiomyocytes exhibited a significantly decreased APD_90_ for corrected-LQTS3/BrS compared with LQTS3/BrS. In resting membrane potentials of atrial type- and ventricular type-iPSC-derived cardiomyocytes, there are significant differences between control and corrected-LQTS3/BrS. This suggests that there might be difference in resting membrane potential among each iPSC clone-derived cardiomyocyte. But importantly, there is no significant difference in resting membrane potentials of atrial type- and ventricular type-iPSC-derived cardiomyocytes between LQTS3/BrS and corrected-LQTS3/BrS. This indicates that LQTS3/BrS associated mutation would affect the APD90 but not resting membrane potential. Patch-clamp analysis revealed that there was no significant difference in peak sodium current between the LQTS3/BrS and corrected-LQTS3/BrS (Fig. 6D,E). The late sodium current and relative late sodium current were significantly decreased in corrected-LQTS3/BrS (Fig. 6F,G). Next, we examined the responses to a ramp voltage protocol, in which membrane potential was gradually changed from +20 to −100 mV following 100-ms deporalizing to +20mV for measuring of late sodium current. The corrected-LQTS3/BrS window current exhibited a smaller peak compared with the LQTS3/BrS (Fig. 6H, Supplementary Figure 2E). These data indicate that the pathological features in LQTS3/BrS iPSC-derived cardiomyocytes were caused solely by the *SCN5A* E1784K mutation.

## Discussion

In the present study, we successfully generated iPSCs from a patient with the *SCN5A* E1784K mutation who exhibited a mixed LQTS3 and BrS phenotype, and differentiated these iPSCs into cardiomyocytes. Functional analysis showed that LQTS3/BrS iPSC-derived cardiomyocytes recapitulated the disease phenotype of LQTS3, which is frequently associated with sudden death in the young, but not that of BrS, which is frequently associated with sudden death in adults. As part of the LQTS3 phenotype, MEA analysis revealed prolonged FPD, whereas patch-clamp experiments showed prolonged APD for ventricular-type, but not nodal- or atrial-type cardiomyocytes. In addition, patch-clamp experiments revealed the presence of late Na^+^ currents, a hallmark biophysical property of LQTS3. These findings are similar to those reported for murine iPSC-derived cardiomyocytes carrying the LQTS3 mutation^31^. The results of expression profiling of Na^+^ channel β-subunits, patch-clamp experiments in heterologous expression systems, and knockdown experiments in iPSC-derived cardiomyocytes suggest that expression of the fetal type Na^+^ channel β-subunit *SCN3B* affects the manifestation of the BrS phenotype in iPSC-derived cardiomyocytes.

**Figure 7:**
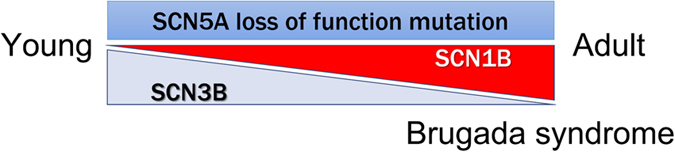
The schematic model of the BrS phenotype and Na^+^ channel β-subunit expression.

We did not detect a reduction in peak Na^+^ currents, an electrophysiological hallmark of BrS, in LQTS3/BrS iPSC-derived cardiomyocytes. In a previous study, reductions in peak Na^+^ currents, a positive shift of the activation curve, and a negative shift of the inactivation curve were reported in a tsA-201 heterologous expression system with the same *SCN5A* E1784K mutation^11^. In the present study, we did not observe any shifts in the activation or inactivation curves in LQTS3/BrS iPSC-derived cardiomyocytes. This apparent discrepancy may be due to the differential intrinsic environment between tsA-201 cells and iPSC-derived cardiomyocytes. In the present study, we found that the expression pattern of Na^+^ channel β-subunits in iPSC-derived cardiomyocytes differed from that in adult hearts, but was very similar to that in fetal hearts. *SCN3B* is the predominant Na^+^ channel β-subunit in fetal hearts and iPSC-derived cardiomyocytes, and *SCN1B* is the predominant form in adult hearts. Those expressions are reversed in association with growth, which would affect the BrS phenotype in adult patients in cell-autonomous manner (Fig. 7). We hypothesized that abundant expression of *SCN3B* masks the BrS-like phenotype in LQTS3/BrS iPSC-derived cardiomyocytes, and investigated this hypothesis with two types of experiments. In one, the tsA-201 heterologous expression system, coexpression of *SCN3B* markedly increased peak Na^+^ currents and shifted the inactivation curve in a positive direction in tsA-201 cells expressing the *SCN5A* E1784 mutation. These SCN3B-dependent changes are concordant with the hypothesis that the expression of *SCN3B* masks the BrS-like phenotype. In the second series of experiments, *SCN3B* siRNA was used to knock down expression of *SCN3B* in iPSC-derived cardiomyocytes. *SCN3B* knockdown shifted the inactivation curve in a negative direction. However, the results of experiments performed in the tsA-201 heterologous expression system and following *SCN3B* knockdown in iPSC-derived cardiomyocytes do not explain the lack of a positive shift of the activation curve. Thus, abundant *SCN3B* expression may be one of the mechanisms underlying the lack of a BrS-like phenotype in iPSC-derived cardiomyocytes.

Mutations in *SCN3B* have been identified in idiopathic ventricular fibrillation, including BrS. It is worth noting that two rare missense mutations in *SCN3B* have been identified in sudden infant death syndrome^32^. An *SCN3B* missense mutation has been identified in a 64-year-old man with a BrS-like electrocardiogram^33^. Animal study showed the importance of the Scn3b to Na channel properties in *Scn3b* knockout mouse heart^34^. Thus, *SCN3B* may be involved in the manifestation of Na^+^ channelopathy. Recently, it was reported that iPSC-derived cardiomyocytes with the *SCN5A* 1795insD mutation manifested both LQTS3- and BrS-like phenotypes with reduced peak Na^+^ currents and the presence of persistent Na^+^ currents^35^. The apparent differences between that study and the present study may be due to the different mutations, racial differences, the method used for cardiomyocyte differentiation, the timing of analysis, and the conditions in the electrophysiological experiments. In the case of the *SCN5A* 1795insD mutation, *SCN3B* knockdown may exaggerate the phenotype of BrS in iPSC-derived cardiomyocytes. Future studies should aim to use iPSCs from many patients with LQTS3 and BrS phenotypes to address these issues. To gain an insight as to how widely *SCN3B* expression affects the phenotype of Na^+^ channelopathy, coexpression experiments for a wide repertoire of *SCN5A* mutations in heterologous expression systems or the generation of iPSC-derived cardiomyocytes with a wide range of *SCN5A* mutations are warranted.

There are some limitations in this study. The iPSC-derived cardiomyocytes showed an immature phenotype. Our iPSC-derived cardiomyocytes were also relatively depolarized, which is one of the immature phenotypes in cardiomyocytes. These embryonic and immature phenotypes of cardiomyocytes are the limitation of the study for disease analysis. Unfortunately, despite on-going attempts to obtain mature iPSC-derived cardiomyocytes resembling adult rod-shape cardiomyocytes, current techniques do not accomplish full maturation. We used one patient with LQTS3/BrS for the current study. And we generated iPSCs by retroviral vectors which integrated host genome, which might affect the disease phenotype. Therefore we generated an isogenic control iPSCs generated by gene correction.

The mechanisms underlying the temporal regulation of disease manifestation in human inheritable disorders are often discussed but are not fully known because of a lack of study methods. It is difficult or impossible to obtain clinical samples from the same patient when they are young and when they are adults. It is generally considered that iPSC-derived cells show an embryonic rather than adult phenotype^14^,^15^. Thus, it is reasonable to assume that iPSC-derived cells will recapitulate the phenotype of early onset diseases, but not those of late-onset diseases. Indeed, there are some reports of the manifestation of specific (early onset) human disease phenotypes by iPSCs^5^,^6^,^7^,^8^. In the present study we showed differential expression of the young-onset LQTS3 and adult-onset BrS phenotypes by iPSC-derived cardiomyocytes, and that intrinsic cell factors underlie the differential manifestation of these two phenotypes. Our findings raise concern regarding the use of iPSC-derived cell models for late-onset hereditary diseases. However, if we are aware of the factors that affect phenotype expression, iPSCs should become a powerful tool with which to elucidate the temporal regulation of the manifestation of genetic diseases.

## Methods

### Patient consent

All subjects provided informed consent for blood testing for genetic abnormalities associated with hereditary LQTS. The isolation and use of patient and control somatic cells was approved by the Ethics Committee of Keio University (approval no. 20–92–5) and was performed only after the patients and controls had provided written informed consent. Our study also conforms with the principles outlined in the Declaration of Helsinki (Cardiovascular Research 1997;35:2–3) for use of human tissue or subjects.

### Fibroblast and iPSC culture

Dermal fibroblasts obtained by dermal biopsy from a patient with LQTS3/BrS and two healthy volunteers were maintained in Dulbecco’s modified Eagle’s medium (DMEM; Sigma-Aldrich, St Louis, MO, USA) with 10% fetal bovine serum (FBS; Nichirei Biosciences, Tokyo, Japan), passaged twice, and used to generate iPSCs, as described below. Human iPSCs were maintained on irradiated mouse embryonic fibroblast (MEF) feeder cells in hiPSC culture medium, consisting of 80% DMEM/F12 (Sigma-Aldrich), 20% KO Serum Replacement (Invitrogen, Carlsbad, CA, USA), 4 ng/mL basic fibroblast growth factor (bFGF; WAKO, Osaka, Japan), 2 mmol/L l-glutamine (Invitrogen), 0.1 mmol/L non-essential amino acids (Sigma-Aldrich), 0.1 mmol/L 2-mercaptoethanol, 100 U/mL penicillin, and 100 μg/mL streptomycin (Invitrogen). The hiPSC medium was changed every 2 days and the cells were passaged using 1 mg/mL collagenase IV (Invitrogen) every 5–7 days. 293FT cells were cultured in DMEM supplemented with 10% FBS (Nichirei Biosciences), 1 × 10^−4 ^M non-essential amino acids (NEAA; Sigma-Aldrich), 2 mmol/L l-glutamine (Invitrogen), 100 U/mL penicillin, and 100 μg/mL streptomycin (Invitrogen).

### Generation of human iPSC

Human iPSCs were established as described previously^23^. Briefly, we used a lentivirus to introduce the mouse *Slc7a1* gene. Transfectants were plated at a density of 2 × 10^5^ cells per 60 mm dish. The following day, *OCT4*, *SOX2*, *KLF4*, and *c-MYC* were introduced by retroviral transfection. Six days later, the cells were harvested and plated at a density of 5 × 10^4^ cells in 100 mm dishes. The cells were cultured for another 20 days. On Day 25, embryonic stem (ES) cell-like colonies were dissociated mechanically and transferred to a 24 well plate on the MEF feeder cells. Further studies using iPSCs used two independent iPSC lines from each patient and healthy control.

### Virus preparation (lentivirus and retrovirus)

Lentiviruses were generated by cotransfecting pLenti6/UbC, encoding the ecotropic retrovirus receptor, the mouse solute carrier family 7, a member 1 (*Slc7a1)*, together with the Virapower packaging mix (pLP1, pLP2, and pLP/VSVG mixture) using Lipofectamine 2000 (Invitrogen), according to the manufacturer’s instructions, into 293FT cells seeded at a density of 4 × 10^6^ cells per 10 cm dish. Lentiviruses were collected 36 h after transfection and filtered through a 0.45 μm cellulose acetate filter. The virus-containing supernatant supplemented with 4 μg/mL polybrene (WAKO) was added to human fibroblasts seeded at a density of 8 × 10^5^ cells per 10 cm dish and incubated for 24 h at 37 °C under 5% CO_2_. The pMXs retroviral vectors consisted of the 5′-long terminal repeat (LTR) and 3′-LTR of Moloney Murine Leukemia Virus (MMLV), encoding the human *OCT4*, *SOX2*, *KLF4*, and *c-MYC* genes, as well as green fluorescence protein to monitor transfection efficacy. PLAT-E packaging cells, which were derived from 293T cells and contained env-IRES-puro and gag-pol-IRES-bs cassettes driven by the EF1-α promoter, were plated at a density of 3.6 × 10^6^ cells per 10 cm dish and incubated overnight at 37 °C. The following day, cells were transfected with pMXs vectors using Fugene 6 transfection reagent (Roche Company, Basel, Switzerland). Forty-eight hours after transfection, the medium was collected and the virus-containing supernatants were filtered through a 0.45 μm filter and supplemented with 4 μg/mL polybrene (WAKO). Equal parts of supernatants containing the four retroviruses were mixed, transferred to fibroblasts expressing the mouse *Slc7a1* gene and seeded at a density of 8 × 10^5^ cells per 10 cm dish, and incubated overnight at 37 °C.

### Genome sequencing

DNA sequencing was used to confirm the presence of the LQT3/BrS mutation in patient-derived iPSCs. Genomic DNA was isolated using a Gentra Puregene Cell Kit (QIAGEN, Valencia, CA, USA) and the region encoding *SCN5A*, including the mutation, was amplified using polymerase chain reaction (PCR) with the following primer set: 5′-GAGCCCAGCCGTGGGCATCCT-3′ (forward) and 5′-GTCCCCACTCACCATGGGCAG-3′ (reverse). The PCR product (310 bp) was electrophoresed on a 1% agarose gel and purified using a Wizard SV Gel and PCR Clean-Up System (Promega, Madison, WI, USA). The purified PCR product was sequenced with original primers.

### Teratoma formation assays

To confirm pluripotency *in vivo*, teratoma formation was assessed in accordance with the Institutional Animal Care and Use Committee of Keio University. Approximately 1 − 2 × 10^6^ iPSCs were injected into the testis of anesthetized immune-compromised NOD-SCID mice (CREA-Japan, Tokyo, Japan). At 10–12 weeks after the injection, mice were euthanized and the teratomas were excised, fixed overnight in formalin, embedded in paraffin, and analyzed by haematoxylin-eosin staining. The mice were anesthetized using a mixture of ketamine (50 mg/kg), xylazine (10 mg/kg), and chlorpromazine (1.25 mg/kg). The adequacy of anesthesia was monitored by heart rate, muscle relaxation, and the loss of sensory reflex responses, i.e., nonresponsive to tail pinching. The investigation conforms with the Guide for the Care and Use of Laboratory Animals published by the US National Institutes of Health (publications number 23–80 revised in 2011) and was approved by university review board in Keio University.

### Immunocytochemistry

Immunostaining was used to analyze the expression of pluripotency or cardiomyocyte markers. Cells were placed on a 35 mm glass-bottomed dish (IWAKI) before being fixed with 4% paraformaldehyde for 30 min at 4 °C. The cells were then rinsed three times with phosphate-buffered saline (PBS) and permeabilized with 0.2% Triton-X 100 in PBS, when needed. The cells were then washed and blocked with Immunoblock (Dainippon Sumitomo Pharma, Osaka, Japan) three times for 5 min each time. Samples were incubated overnight at 4 °C with each of the primary antibodies. For iPSCs, these antibodies were anti-Nanog (1:200 dilution; ab21624; Abcam, Cambridge, UK), anti-Oct4 (1:100 dilution; sc-5279; Santa Cruz Biotechnology, Dallas, TX, USA), anti-SSEA3 (1:200 dilution; MAB4303; EMD Millipore, Billerica, MA, USA), anti-SSEA4 (1:200 dilution; MAB4304; EMD Millipore), anti-Tra1-60 (1:200 dilution; MAB4360; EMD Millipore), and anti-Tra1–81 (1:200 dilution; MAB4381; EMD Millipore); for cardiomyocytes, the primary antibodies were anti-cTnT (1:200 dilution; MS-295-PABX; Thermo Fisher Scientific, Waltham, MA, USA) and anti-actinin (1:250 dilution; A7811; Sigma-Aldrich). Following incubation with primary antibodies, samples were incubated at room temperature for 1 h with the following secondary antibodies: Alexa Fluor 488 goat anti-rat IgG (1:200 dilution; A11006; Invitrogen), Alexa Fluor 594 chicken anti-mouse IgG (1:200 dilution; A21201; Invitrogen), Alexa Fluor 488 goat anti-rat IgM (1:200 dilution; A21212; Invitrogen), Alexa Fluor 594 goat anti-mouse IgM (1:200 dilution; A21044; Invitrogen), Alexa Fluor 488 chicken anti-mouse IgG (1:200 dilution; A21200; Invitrogen), Alexa Fluor 594 goat anti-rabbit IgG (1:200 dilution; A11037; Invitrogen), and Alexa Fluor 555 goat anti-mouse IgG1 (1:200 dilution; A21127; Invitrogen). After cells had been washed by PBS, samples were mounted using Vectashield Hard Set Mounting Medium with 4′,6′-diamidino-2-phenylindole (Vector Laboratories, Burlingame, CA, USA). Images were obtained using a ×10 objective lens (NA = 0.45) on a fluorescence microscope (BZ-9000; Keyence, Osaka, Japan) or a ×63 (NA = 1.2) lens installed on a confocal microscope (LSM 510 Duo; Carl Zeiss, Oberkochen, Germany).

### Differentiation of iPSCs into cardiomyocytes

Harvested human iPSC colonies with collagenase type IV were washed on a 100 μm Cell Strainer (352360; Becton, Dickinson and Company, Franklin Lakes, NJ, USA) to avoid contamination with MEFs. Colonies were transferred to non-coated dishes (AGC TECHNO GLASS, Shizuoka, Japan) in cardiomyocyte differentiation medium, comprised of minimum essential medium (MEM; 12000–022; Invitrogen) with 1× GlutaMAX containing 20% FBS, 1 mM NEAA, 0.1 mM 2-mercaptoethanol, 100 U/mL penicillin, and 100 μg/mL streptomycin. The medium was exchanged for fresh medium the following day and then weekly thereafter. After 30 days, beating embryoid bodies (EBs) were harvested for use in other experiments. The beating EBs were transferred to microcentrifuge tubes in EB dissociation solution (80% ADS buffer, which contains 116 mmol/L NaCl, 5.4 mmol/L KCl, 1 mmol/L NaH_2_PO_4_, 0.8 mmol/L MgSO_4_, 5.5 mmol/L glucose, 20 mmol/L HEPES [pH adjusted to 7.35 with NaOH], 10% of 1 g/L collagenase type IV in DMEM/F12 and 10% of 2.5% Trypsin [Invitrogen]). The EBs were dissociated to single cells using a microstirring bar at 37 °C for 30 min. The cells were then transferred to fibronectin-coated dishes (F1141; Sigma-Aldrich).

### Transfection of siRNA

Cardiomyocytes were reverse transfected with 30 pmol siRNAs (4390843 as a negative control; 4392420 for *SCN3B*; Applied Biosystems) using 5 μL Lipofectamine RNAiMAX (Invitrogen) according to the manufacturer’s instructions. Three days after transfection, patch-clamp analysis was performed.

### Multielectrode array recordings

Randomly chosen iPSCs from two healthy volunteers (control) and the patient with LQTS3/BrS were used for electrophysiological characterization. Two independent iPSC lines were used from the patient. The electrophysiological properties of beating EBs were assessed using a multielectrode array (MEA) recording system (Multichannel Systems, Reutlingen, Germany). The beating EBs were transferred to fibronectin-coated MEA plates. The extracellular potential was recorded in DMEM/HEPES (D5796; Sigma-Aldrich) at 37 °C at 10 kHz temporal resolution. The electrograms recorded were used to determine field potential duration (FPD). This parameter corresponds to the action potential duration (APD) and reflects the QT interval. The estimated FPD was defined as the time interval between the initial deflection of the field potential and the time of the maximum of the next wave. FPD measurements were corrected (cFPD) for the rate of beating of the EBs using Fridericia’s formula: cFPD = FPD/3√(RR interval).

### Electrophysiological analysis using a heterologous expression system

Na^+^ currents were recorded using the whole-cell patch-clamp technique, as described previously^22^. Briefly, the human tsA-201 cell line was transiently transfected with wild-type (WT) or E1784K human *SCN5A* plasmid using Lipofectamine (Invitrogen) in combination with a bicistronic plasmid (pCD8-IRES) encoding CD8 and a human Na^+^ channel β-subunit (either *SCN1B*, *SCN2B*, or *SCN3B*) to visually identify cells expressing heterologous β-subunits with Dynabeads (M-450 CD8; Dynal). Electrophysiological measurements were obtained 24–72 h after transfection. The holding potential was −120 mV. The composition of the bath solution was (in mmol/L): NaCl 145; KCl 4; CaCl_2_ 1.8; MgCl_2_ 1; HEPES 10; and glucose 10, pH 7.35 (adjusted with NaOH). The composition of the pipette solution (intracellular solution) was (in mmol/L): NaF 10; CsF 110; CsCl 20; EGTA 10; HEPES 10, pH 7.35 (adjusted with CsOH).

To determine activation parameters and peak current density, the I-V relationship was fitted to the Boltzmann equation:


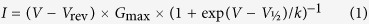


where *I* is the peak Na current during the test pulse potential *V*. The parameters estimated by the fitting are the reversal potential (*V*_rev_), maximum conductance (*G*_max_), voltage for half-activation (*V*_½_), and the slope factor (*k*). Steady state inactivation was fitted to the following Boltzmann equation:


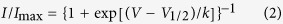


to determine the membrane potential for *V*_½_ and *k*. Recovery from inactivation was fitted using biexponential functions:


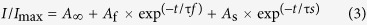


where *I*_max_ is the maximum peak Na^+^ current, *A*_∞_ is a constant value, *A*_f_ and *A*_s_ are the fractions of the fast and slow recovering components, respectively, and τ_f_ and τ_s_ are the time constants of the fast and slow recovering components, respectively.

### Quantitative reverse transcription–polymerase chain reaction

Human RNA samples were purchased from TAKARA Bio (Shiga, Japan). Total RNA was purified using Trizol reagent (Invitrogen) and was treated with the Turbo DNA-free kit (Ambion) to remove genomic DNA. Total RNA (1 μg) was used for reverse transcription with SuperScript II Reverse Transcriptase (Invitrogen) according to the manufacturer’s instructions. Quantitative polymerase chain reaction (PCR) was performed using Taqman Probes (Applied Biosystems) and analyzed with the 7500 Real-Time PCR System (Applied Biosystems). The Taqman probes used were as follows: Hs00962350_m1 for SCN1B, Hs00394952_m1 for SCN2B, Hs01024483_m1 for SCN3B, Hs00545394_m1 for SCN4B, Hs00153998_m1 for ANK2B, Hs00165960_m1 for TNNT2 and Hs02758991_g1 for GAPDH. Quantitative RT-PCR for *SCN1B beta* and *GAPDH* was performed using Perfect Real Time SYBR *Premix Ex Taq* II kit (Takara, Japan) using primer set; GGTGCCTTCTGTCTCTGAGC and AGAATGGCTCAAACCACACC, CAGAACATCATCCCTGCCTCTAC and TTGAAGTCAGAGGAGACCACCTG. Samples were cycled 40 times using a 7500 Real Time PCR System (Applied Biosystems) and cycle conditions were as follows: 2 min at 50 °C 10 min. at 95 °C followed by 40 cycles of 15 sec. at 95 °C and 60 sec. at 60 °C. Cycle threshold was calculated under default settings by real-time sequence detection software (Applied Biosystems). The expression levels of *SCN1B* were normalized to that of *GAPDH*.

### Patch-clamp experiments (iPSC-derived cardiomyocytes)

Whole-cell or perforated patch-clamp recordings of human cardiomyocytes generated from iPSCs were made using an Axopatch 200B (Molecular Devices, Sunnyvale, CA, USA) and an inverted microscope equipped with differential interface optics (TE2000-U; Nikon, Tokyo, Japan). Glass pipettes were prepared from borosilicate glass (Harvard Apparatus, Holliston, MA, USA) using a micropipette puller (Model P-97; Sutter Instruments, Novato, CA, USA). Voltage-clamp measurements were obtained using an extracellular solution consisting of 135 mmol/L NaCl, 5.4 mmol/L CsCl, 0.53 mmol/L MgCl_2_, 2 mmol/L CaCl_2_, 5 mmol/L HEPES–NaOH, 5.5 mmol/L glucose and 0.1 mmol/L CoCl_2_ (pH 7.4 at 25 °C) and a pipette solution consisting of 130 mM CsCl, 2 mmol/L MgCl_2_, 5 mmol/L ATP–2Na, 10 mmol/L EGTA, 20 mmol/L TEA–Cl and 10 mmol/L HEPES (pH adjusted to 7.3 with CsOH). To measure the amplitude of the Na^+^ peak and late currents, the whole-cell configuration of the patch clamp was used. Cells were held at −100 mV and depolarized to −10 mV for 200 ms at a rate of 0.33 Hz. The current–voltage (I-V) relationship was measured using the following protocol: 200-ms depolarizing steps from a holding potential of −100 mV to potentials ranging from −70 to +20 mV in 10-mV increments. Action potentials were recorded in the perforated patch-clamp configuration. Current-clamp recordings were obtained in normal Tyrode’s solution (composition [in mmol/L]: NaCl 135; NaH_2_PO_4_ 0.33; KCl 5.4; CaCl_2_ 1.8; MgCl_2_ 0.53; glucose 5.5; HEPES 5; pH 7.4 at 35 °C) using a pipette solution with the following composition (in mmol/L): d,l-aspartic acid potassium 110; KCl 30; Mg-ATP 5; Na_2_-phospho-creatine 5; EGTA 10; CaCl_2_ 1; HEPES 5 (pH adjusted to 7.25 with KOH). Just before use, 0.3 g/L amphotericin B was added to the pipette solution to perforate the cell membrane. Ventricular-type action potentials are distinguished by the presence of a marked plateau phase and an APD90/APD50 ratio up to 1.3^5^. Atrial-type action potentials are distinguished by the presence of a triangle shape with a higher APD90/50 ratio between 1.3 and 1.6. Nodal action potentials are distinguished by less negative maximum diastolic potential (MDP), slower upstroke (dV/dtmax) and smaller AP amplitude (APA). The time course of inactivation was fit with a biexponential function: 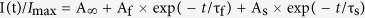, where A_∞_ is a constant value, A_f_ and A_s_ are fractions of fast and slow inactivating components, and τ_f_ and τ_s_ are the time constants of fast and slow inactivating components, respectively.

### Preparation of HDAdVs for gene targeting

HDAdVs were constructed and prepared as previously described^36^,^37^. First, the 5′ homologous arms of the WT *SCN5A* gene were amplified using LA-primer-F and LA-primer-R, and the 3′ homologous arm was amplified using RA-primer-F and RA-primer-R. The wild-type gene was amplified from iPS line 4F16 genome. Using PL452 plasmid, which contains a fragment of the PGK-*Neo*-bGHpA cassette sandwiched by loxP sites, cassette was inserted into each amplified *SCN5A* gene between exons 27 and 28. The fragment was then transferred to the BAC clone^38^ (RP11-356A22, purchased form invitrogen) by homologous recombination in *E*. *coli*. Next, the 24.3-kb region (consists of 12.8-kb of 5′-homology arm, 1.9-kb of *Neo* cassette and 9.6-kb of 3′-homology arm) was inserted into the HDAdV plasmid by homologous recombination in *E-coli*^36^. The constructs were linearized and propagated by serial passage in 293FLPe cells with the addition of the FL helper virus. The viruses were purified by ultracentrifuge with CsCl gradient^39^.

### Gene targeting

iPSCs from patients were dissociated to single cells with accutase (funakoshi, Tokyo, Japan). iPSCs were suspended into 100 micro L of conditioned medium containing bFGF and Y-27632 and infected with HDAdVs for 1 hr at room temperature. Selection with 50 micro g/ml of G418 (WAKO) was performed. After the appearance of colonies resistant to G418, they were picked up and additional selection with 200 nmol/L of FIAU (WAKO) was performed. The genomic DNA of the drug-resistant clones was screened by genomic sequence. To remove the *Neo* cassette, the cells were dissociated into single cells once again and transfected with pCAG-CreN (kindly provided by Dr. Konosuke Mitani, Saitama medical university) using fugene HD (Promega). The removal of the *Neo* cassette was confirmed by G418 sensitivity and PCR using Neo-test-primers a-f. Each clone of iPSCs was sequenced and checked the mutation was properly corrected.

LA-primer-F: gggaattcgatgtagaagatgggcctag

LA-primer-R: ttgtcgactgaggcttatctggttgggc

RA-primer-F: aagcggccgcgatggctataaagtgctcgg

RA-primer-R: caggatccttgggttctcaggccagagg

Neo-test-primer a: ctaggcccatcttctacatc

Neo-test-primer b: atagagctgtcctaggccca

Neo-test-primer c: cactcccactgtcctttcct

Neo-test-primer d: ggggccaccaaagaacggag

Neo-test-primer e: gctgggtgggcaagatacta

Neo-test-primer f: agggagcactgatttctggg

Genomic sequence primer outside of targeting vector g: tgtgtgcactgatttcccaa

### Statistical analyses

Values are presented as the mean ± SEM. The significance of differences between two means was evaluated with unpaired *t*-test. Chi-squared analysis was used for comparisons between two groups. Comparisons between more than three groups were made by ANOVA followed by Bonferroni’s multiple comparison test. *P* < 0.05 was considered significant.

## Additional Information

**How to cite this article**: Okata, S. *et al*. Embryonic type Na^+^ channel β-subunit, *SCN3B* masks the disease phenotype of Brugada syndrome. *Sci. Rep.* **6**, 34198; doi: 10.1038/srep34198 (2016).

## Supplementary Material

Supplementary Information
