# Supplementary material for: Embryonic type Na+ channel β-subunit, SCN3B masks the disease phenotype of Brugada syndrome
Source: Sci Rep. 2016 Sep 28;6:34198. doi: 10.1038/srep34198 (PMC5039759; doi:10.1038/srep34198)
Supplement: Supplementary Information [file srep34198-s1.pdf]

Supplementary Information

Embryonic type Na^+ channel β-subunit, *SCN3B* masks the disease phenotype of Brugada syndrome

Shinichiro Okata[1, 2], MD, PhD, Shinsuke Yuasa* [1], MD, PhD, Tomoyuki Suzuki[1, 3], MD, PhD, Shogo Ito[1], MD, Naomasa Makita[4], MD, PhD, Tetsu Yoshida[5], PhD, Min Lin[2], PhD, Junko Kurokawa[2], PhD, Tomohisa Seki[1], MD, PhD, Toru Egashira[1], MD, PhD, Yoshiyasu Aizawa[1], MD, PhD, Masaki Kodaira[1], MD, PhD, Chikaaki Motoda[1], MD, Gakuto Yozu[1], MD, Masaya Shimojima[1], MD, Nozomi Hayashiji[1], PhD, Hisayuki Hashimoto[1], MD, PhD, Yusuke Kuroda[1, 3], MD, Atsushi Tanaka[1], MD, PhD, Mitsushige Murata[1, 6], MD, PhD, Takeshi Aiba[7], MD, PhD, Wataru Shimizu[8], MD, PhD, Minoru Horie[9], MD, PhD, Kaichiro Kamiya[3], MD, PhD, Tetsushi Furukawa[2], MD, PhD, Keiichi Fukuda[1], MD, PhD

1: Department of Cardiology, Keio University School of Medicine, Japan.
2: Department of Bio-informational Pharmacology, Medical Research Institute, Tokyo Medical and Dental University, Japan.
3: Department of Cardiovascular Research, Research Institute of Environmental Medicine, Nagoya University, Japan.
4: Department of Molecular Pathophysiology-1, Nagasaki University Graduate School of Biomedical Sciences, Nagasaki, Japan.
5: Division of Gene Therapy, Research Center of Genome Medicine, Saitama Medical University, Japan.
6: Department of Laboratory Medicine, Keio University School of Medicine, Tokyo, Japan.
7: Division of Arrhythmia and Electrophysiology, Department of Cardiovascular Medicine, National Cerebral and Cardiovascular Center, Osaka, Japan.
8: Department of Cardiovascular Medicine, Nippon Medical School, Tokyo, Japan.
9: Department of Cardiovascular Medicine, Shiga University of Medical Science, Shiga, Japan.

Supplementary Figure 1. Differentiation ability of LQTS3/BrS iPSCs

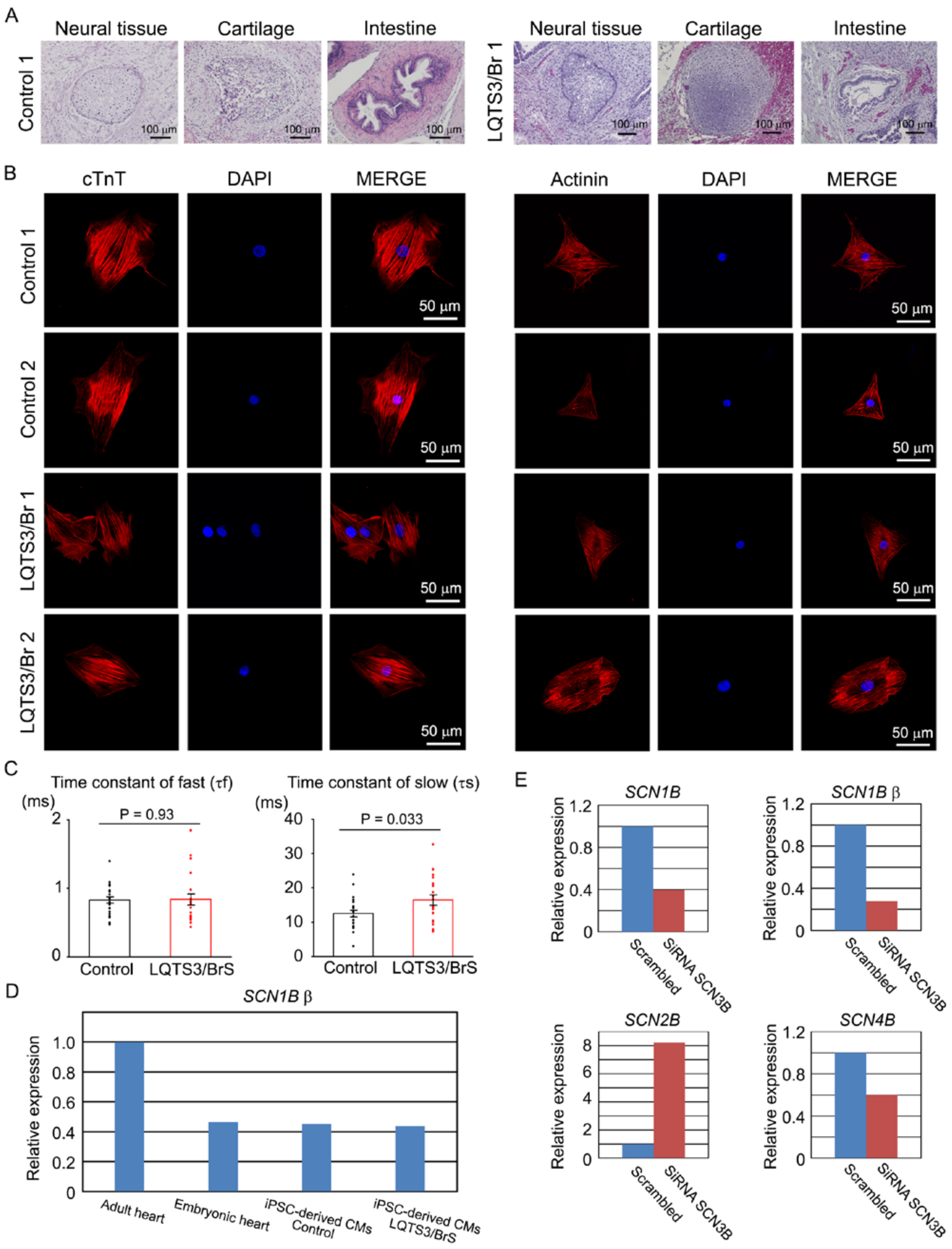

A. Teratoma formation following injection of LQTS3/BrS-iPSCs in immune-compromised NOD-SCID mice. Sections of teratomas were stained with hematoxylin and eosin, and tissues representative of all three germ layers were observed. Scale bars, 100 μm. **B.** Immunofluorescence staining for cardiomyocyte markers (cardiac troponin T [cTnT] and actinin) in two control and two LQTS3/BrS iPSC-derived cardiomyocytes. DAPI, 4′,6′-diamidino-2-phenylindole. **C.** Time constants of current decay of Na1 current in control- and LQTS3/BrS-iPSC-derived cardiomyocytes, measured by macropatch. Current decay at each potential was fit with a biexponential function; time constants of fast (τf) and slow (τs) components are shown. **D.** Quantitative RT-PCR analyses for *SCN1Bβ* in adult human heart (mixed sample from three men aged 30–39 years), embryonic heart (mixed human sample from 34 male and female embryos at 12–31 weeks gestation), control-iPSC-derived-cardiomyocytes and LQTS3/BrS-iPSC-derived cardiomyocytes. **E.** Quantitative RT-PCR analyses of *SCN1B, SCN1Bβ, SCN2B* and *SCN4B* in iPSC-derived cardiomyocytes transfected with either scrambled siRNA or siRNA for *SCN3B.*

Supplementary Figure 2. Electrophysiological features of corrected-LQTS3/BrS iPSC-derived cardiomyocytes

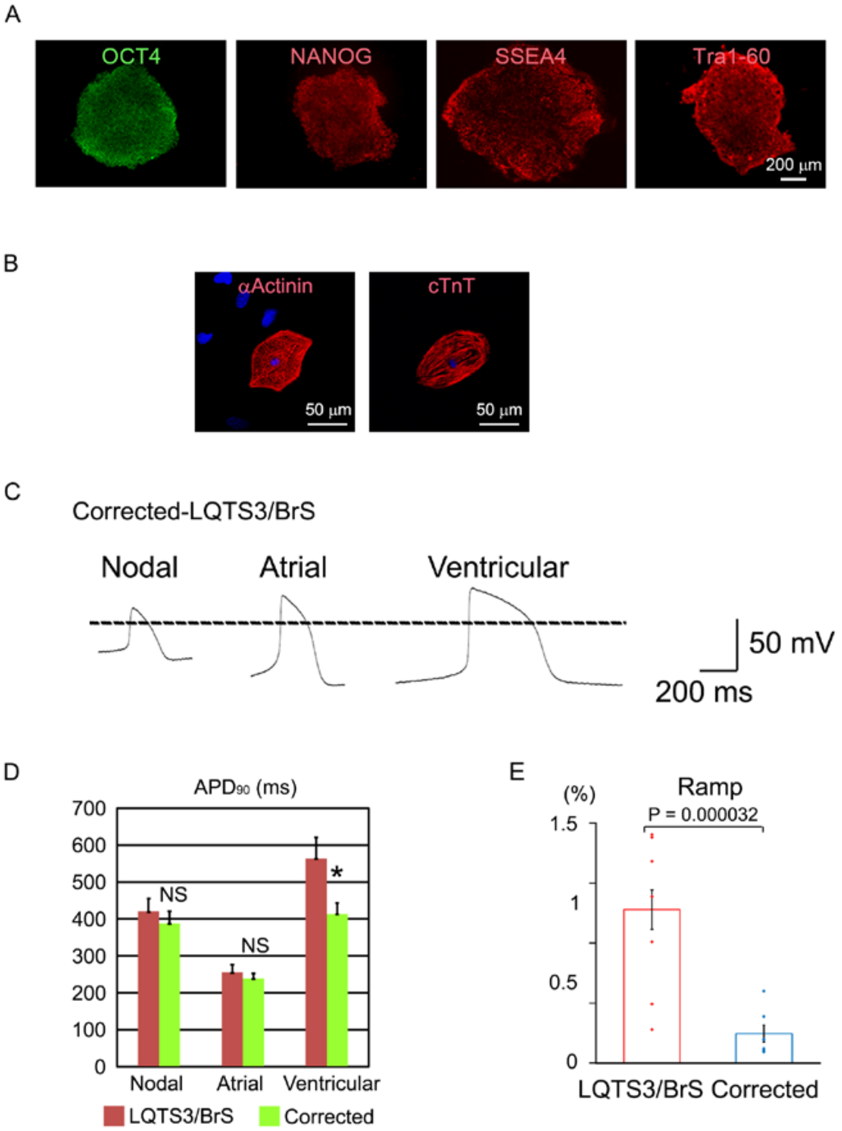

A. Immunofluorescence staining for stem cell markers (OCT4, NANOG, SSEA4 and Tra1-60) in corrected-LQTS3/BrS iPSC colonies. **B.** Immunofluorescence staining for cardiomyocyte markers (αActinin and cardiac troponin T [cTnT]) in corrected-LQTS3/BrS iPSC-derived cardiomyocytes. **C.** Representative action potentials of corrected-LQTS3/BrS iPSC-derived cardiomyocytes showing nodal-, atrial- and ventricular-type morphology. The dashed line indicate 0 mV. **D.** Statistical parameters of action potential duration at 90% repolarization (APD_{90}) obtained from LQTS3/BrS and corrected-LQTS3/BrS iPSC-derived cardiomyocytes exhibiting nodal- (n = 19 and 11, respectively), atrial- (n = 4 and 8, respectively) and ventricular-type (n = 7 and 10, respectively) morphology. Where appropriate, data are given as the mean ± SEM. *$P < 0.05$ compared with control. **E.** The peak of ramp currents normalized to the peak current recorded in the same cell in LQTS3/BrS (n= 13) and corrected-LQTS3/BrS iPSC-derived cardiomyocytes (n= 9).

Supplementary Table. Electrophysiological characteristics of iPSC-derived cardiomyocytes

Nodal type-iPSC-derived cardiomyocytes:

	Maximum upstroke velocity (V/S)	APD 50 (ms)	APD 90 (ms)	Over shoot (mv)	Amplitude (mv)	Resting potential (mV)	n
control	2.9 ± 0.8	134.1 ± 20.7	354.5 ± 53.3	19.7 ± 2.7	56.2 ± 4.9	-36.5 ± 2.9	6
LQTS3/BrS	3.0 ± 2.4	174.0 ± 19.7	419.9 ± 30.7	23.9 ± 1.6	63.2 ± 3.2	-39.3 ± 2.1	19
corrected	2.8 ± 0.7	153.7 ± 22.2	355.8 ± 33.5	25.7 ± 3.3	61.2 ± 4.7	-35.4 ± 2.1	11

p value (Bonferroni)

	Maximum upstroke velocity (V/S)	APD 50 (ms)	APD 90 (ms)	Over shoot (mv)	Amplitude (mv)	Resting potential (mV)
control vs. LQTS3/BrS	1	0.72363	0.98081	1	0.89955000	1
control vs. corrected	1	1	1	0.71073	1.00000000	1
LQTS3/BrS vs. corrected	1	1	0.70985	1	1	0.70199

Atrial type-iPSC-derived cardiomyocytes:

	Maximum upstroke velocity (V/S)	APD 50 (ms)	APD 90 (ms)	Over shoot (mv)	Amplitude (mv)	Resting potential (mV)	n
control	39.1 ± 12.8	108.8 ± 12.4	237.4 ± 16.4	28.3 ± 3.6	98.3 ± 2.4	-69.9 ± 5.6	6
LQTS3/BrS	10.0 ± 2.6	127.9 ± 7.2	255.0 ± 21.0	30.1 ± 0.8	89.0 ± 1.4	-59.0 ± 1.6	4
corrected	7.6 ± 1.1	141.2 ± 16.8	244.3 ± 14.3	34.3 ± 2.3	89.8 ± 3.8	-55.4 ± 1.1	8

p value (Bonferroni)

	Maximum upstroke velocity (V/S)	APD 50 (ms)	APD 90 (ms)	Over shoot (mv)	Amplitude (mv)	Resting potential (mV)
control vs. LQTS3/BrS	0.08107	1	1	1	0.18203000	0.17821
control vs. corrected	0.01928	0.38954	1	0.36988	0.12422000	0.01703
LQTS3/BrS vs. corrected	1	1	1	0.96459	1	1

Ventricular type-LQT3/BrS-iPSC-derived cardiomyocytes:

	Maximum upstroke velocity (V/S)	APD 50 (ms)	APD 90 (ms)	Over shoot (mv)	Amplitude (mv)	Resting potential (mV)	n
control	15.8 ± 3.8	247.3 ± 30.5	416.7 ± 24.0	34.4 ± 2.5	99.3 ± 3.9	-65.0 ± 4.0	10
LQTS3/BrS	21.8 ± 10.8	326.1 ± 74.5	563.7 ± 57.1	37.4 ± 1.8	97.4 ± 4.0	-60.0 ± 2.8	7
corrected	8.6 ± 1.6	250.9 ± 40.0	418.0 ± 29.6	39.3 ± 1.2	93.1 ± 2.9	-53.8 ± 1.9	10

p value (Bonferroni）

	Maximum upstroke velocity (V/S)	APD 50 (ms)	APD 90 (ms)	Over shoot (mv)	Amplitude (mv)	Resting potential (mV)
control vs. LQTS3/BrS	1	0.7474	0.02809	1	1.00000000	0.88136
control vs. corrected	1	1	1	0.31901	0.62100000	0.04073
LQTS3/BrS vs. corrected	0.34571	0.81293	0.02979	1	1	0.57432
